# Capturing How the Accelerometer Measured Physical Activity Profile Differs in People with Diabetic Foot Ulceration

**DOI:** 10.3390/s24154875

**Published:** 2024-07-27

**Authors:** Liam Neal, Matthew McCarthy, Paddy Dempsey, Francesco Zaccardi, Rachel Berrington, Emer M. Brady, Charlotte L. Edwardson, Frances Game, Andrew Hall, Joseph Henson, Kamlesh Khunti, Bethany Turner, David Webb, Melanie J. Davies, Alex V. Rowlands, Tom Yates

**Affiliations:** 1 Diabetes Research Centre, University Hospitals of Leicester NHS Trust, Leicester LE5 4PW, UK; lgn2@leicester.ac.uk (L.N.);; 2 NIHR Applied Research Collaboration East Midlands (ARC EM), University of Leicester, Leicester LE1 7RH, UK; 3 NIHR Leicester Biomedical Research Centre, University of Leicester, Leicester LE5 4PW, UK; 4 School of Exercise & Nutrition Sciences, Deakin University, Burwood 3125, Australia; 5 University Hospitals of Leicester NHS Trust, Leicester LE1 5WW, UK; 6 Department of Cardiovascular Sciences, University of Leicester, Leicester LE1 7RH, UK; 7 University Hospitals of Derby and Burton NHS Foundation Trust, Derby DE22 3NE, UK; 8 The Hanning Sleep Laboratory, University Hospitals of Leicester NHS Trust, Leicester LE5 4PW, UK

**Keywords:** diabetic foot ulcers, physical activity, accelerometry

## Abstract

Diabetic Foot Ulcers (DFUs) are a major complication of diabetes, with treatment requiring offloading. This study aimed to capture how the accelerometer-assessed physical activity profile differs in those with DFUs compared to those with diabetes but without ulceration (non-DFU). Participants were requested to wear an accelerometer on their non-dominant wrist for up to 8days. Physical activity outcomes included average acceleration (volume), intensity gradient (intensity distribution), the intensity of the most active sustained (continuous) 5–120 min of activity (MX_CONT_), and accumulated 5–120 min of activity (MX_ACC_). A total of 595 participants (non-DFU = 561, DFU = 34) were included in the analysis. Average acceleration was lower in DFU participants compared to non-DFU participants (21.9 mg [95%CI:21.2, 22.7] vs. 16.9 mg [15.3, 18.8], *p* < 0.001). DFU participants also had a lower intensity gradient, indicating proportionally less time spent in higher-intensity activities. The relative difference between DFU and non-DFU participants was greater for sustained activity (MX_CONT_) than for accumulated (MX_ACC_) activity. In conclusion, physical activity, particularly the intensity of sustained activity, is lower in those with DFUs compared to non-DFUs. This highlights the need for safe, offloaded modes of activity that contribute to an active lifestyle for people with DFUs.

## 1. Introduction

Regular engagement in physical activity and reductions in sedentary time are important in the glycaemic management and overall health of people with diabetes [1]. Diabetic Foot Ulcers (DFUs) are a major complication of diabetes and cause a considerable health burden and reduction in life expectancy [2,3]. The estimated lifetime risk of developing DFUs in those who have been diagnosed with type 1 or type 2 diabetes is between 19 and 34% [4,5]. Offloading mainly via therapeutic footwear and limiting weight-bearing activity is the frontline management for DFUs, with the aim of reducing foot pressures and tissue stress, aiding healing, preventing adverse outcomes, and preventing further ulceration [6].

Physical activity and exercise are key components of a healthy lifestyle and are recommended to patients with diabetes as a tool to improve their condition and overall health [1,7]. However, foot complications which can reside alongside diabetes should also be considered [1,7,8]. Despite the importance of recommending physical activity to patients with diabetes, this may not be appropriate in those with DFUs, even when correct offloading footwear is worn [9]. For example, a reduction in weight-bearing activity has commonly been prescribed to aid healing [9]. These activity recommendations and subsequent reductions in mobility due to offloading have the potential to be detrimental to DFU patients’ physical activity levels and, consequently, their health status, which is already suboptimal. The offloading treatments, activity advice, underlying conditions of DFUs, and comorbidities, including neuropathy of patients, all contribute to an adverse environment for undertaking physical activity.

Limited engagement in physical activity and increased sedentary time is, therefore, assumed in this population. However, few data exist on device-based measured activity levels in this population [10,11,12,13], limiting understanding of how patterns of activity may be impacted. A recent review provided some initial insight suggesting that those with DFUs undertake 4248 steps/day [14]. However, previous studies that have focused on steps taken per day do not take into account the intensity of movement, which has particular relevance to DFUs, or other dimensions of daily 24 h movement profiles, such as sleep, which is increasingly recognised in the management of type 2 diabetes [15,16]. Recent advancements in accelerometery allow greater insight into the profile of 24 h physical behaviours. This includes the use of MX metrics; these metrics report the intensity (acceleration) of physical activity above which the most active (X) minutes are accumulated throughout the day [17]. Greater values represent more intense physical activity within the specified duration. These metrics avoid the limitations of cut-point approaches that are commonly used to collapse accelerometer data into pre-defined intensity categories prior to analysis. This approach maintains the continuous nature of the variable; thus, multiple different cut-points can be used to interpret the data [17]. This study, therefore, aimed to provide a more detailed understanding of the physical activity intensity and sleep profile, assessed with accelerometers, for individuals with active DFUs. This examination will enable exploration of which aspects of the 24 h movement profiles are most compromised in individuals with DFUs relative to those with diabetes but without ulceration. This study will help inform clinical practise, provide novel insights into this population, and act as a stimulus for future research.

## 2. Materials and Methods

Data used in this study were collected as part of the CODEC study (Chronotype of Patients with Type 2 Diabetes and Effect on Glycaemic Control; Clinical Trial Registry Number: NCT02973412). The study process is outlined below, and a detailed description of the protocol is available elsewhere [18]. Full ethical approval by the Institutional Research Ethics Committee (16/WM/0457) was received. All participants provided written informed consent.

The inclusion criteria for the CODEC study included a willingness and ability to give informed consent for participation in the study; established T2DM (>6 months since diagnosis); male or female; aged 18 to 75 years inclusive; a body mass index (BMI) less than or equal to 45 kg/m^2^ inclusive; no known sleep disorders except OSA; glycated haemoglobin (HbA1c) up to and below 10% (86 mmol/mol); and proficient in the English language.

Adult participants (18–75 years) diagnosed with type 2 diabetes mellitus with and without DFUs were recruited from secondary care sites within the East Midlands between December 2016 and June 2019. During this period, recruitment sites were expanded to include secondary care foot clinics. Participants with currently unhealed DFUs who were receiving ongoing treatment were identified by participating physicians and invited to take part. Where available, the referring physician also provided the SINBAD score. The SINBAD score is used as a marker of ulcer severity and is scored using six criteria: site, ischaemia, neuropathy, bacterial infection, area, and depth. It is scored 0–6, with increased scores reflecting greater severity [19]. Demographic and anthropometric data and medical history were collected during a single data collection visit. A subset of participants wore an accelerometer (GENEActiv, Activinsights Ltd., Kimbolton, UK), initialised to collect data at 100 Hz, on their non-dominant wrist 24 h/day for up to 8 days. 

### Accelerometer Procession and Statistical Analysis

Data were downloaded using GENEActiv PC software version 3.2. The 100 Hz GENEActiv.bin files were processed using R-package GGIR version 1.8–1 (http://cran.r-project.org, accessed 20 January 2019) [20]. Briefly, the processing in GGIR involved the following steps: (1) auto-calibration of the signal according to local gravity; (2) detection of non-wear times and calculation of the average magnitude of dynamic acceleration corrected for gravity, averaged over 5 s epochs and expressed in milli-gravitational units (Euclidean Norm minus 1 g with negative values rounded up to zero, ENMO, mg); (3) detection of sustained inactivity periods; (4) detection of the sleep window; (5) labelling of sustained inactive periods as sleep or daytime sustained inactivity; and (6) merging of physical activity and sleep information for participants. Non-wear was imputed using the default setting; that is, invalid data were imputed by the average at similar time points on different days of the week. Participants were excluded if post-calibration error was >0.01 g (10 mg), they had <3 days of valid wear (defined as >16 h per day), or if wear data were not present for each 15 min period of the 24 h cycle.

The outcomes generated are described in Table 1. In brief, they were: average acceleration over the 24 h day (reflective of the volume or overall physical activity level in mg); intensity gradient (a measure of distribution of activity intensities across the 24 h day [17]); sleep duration (time accumulated in minutes sleeping during sleep window); time spent inactive daily (total and in prolonged bouts (>30 min)), time spent in light-intensity activity, and time spent in moderate-to-vigorous physical activity (MVPA) in 1 min intervals. The following acceleration thresholds, based on previously defined device-specific thresholds, were used to classify activity intensity: inactivity: <40 mg [21,22] and MVPA: >100 mg [23]. To explore whether there are differences in the way activity is accumulated between those with and without DFU, two additional types of metrics were extracted: (1) the intensity of the most active accumulated 5, 10, 30, 60, and 120 min (M5_ACC_-M120_ACC_) throughout each 24 h period and (2) the intensity of the most active continuous 5, 10, 30, 60, and 120 min (M5_CONT_-M120_CONT_) bout of the 24 h period. The M5_ACC_-120_ACC_ represents the acceleration above which a person’s most active 5, 10, 30, 60, and 120 min are accumulated across the day. The M5_CONT_-120_CONT_ metric represents the acceleration level above which the most active continuous 5, 10, 30, 60, and 120 min ibout of the day was accumulated. This measure indicated that 75% of the most active continuous periods lasting 5, 10, 30, 60, and 120 min had an acceleration above a certain threshold, i.e., if the M30_CONT_ > 100 mg, then the majority of that 30 min period was spent in MVPA (>100 mg), with less than 25% of the time dropping below this threshold; for example, this could include stopping at traffic lights or tying shoelaces. The M5_CONT_-M120_CONT_ and M5_ACC_-M120_ACC_ metrics were included to allow a fuller description of physical activity profiles than those enabled through traditional time-based and cut-point-based metrics, especially in relation to DFUs where the maximum intensity of movement was hypothesised to be particularly impacted. The sleep window and sleep duration within the sleep window were calculated within GGIR using automated sleep detection (HDCZA sleep detection algorithm) [24,25]. The average across all valid days was reported for all outcomes. 

Generalised linear regression models were used to generate estimated marginal means (with 95% CI) adjusted for age, sex, BMI, ethnicity, presence of cardiovascular disease, diabetes duration, and accelerometer wear time. For non-normally distributed data, models using a gamma distribution were used where it improved model fit (AIC criterion). Those with missing covariate data were not included in the analysis and pairwise deletion method was used for any missing accelerometer data. All analyses were conducted using SPSS v.26.3.

## 3. Results

In total, 595 participants had valid accelerometer files and complete covariate data (non-DFU = 561, DFU = 34). Demographic, anthropometric, and accelerometer data are presented in Table 2. The mean accelerometer wear times were 6.9 days (±0.4) and 6.8 days (±0.7) for those with non-DFUs and DFUs, respectively. The median SINBAD score for those with DFUs was 1 (IQR: 1–3).

**Figure 1:**
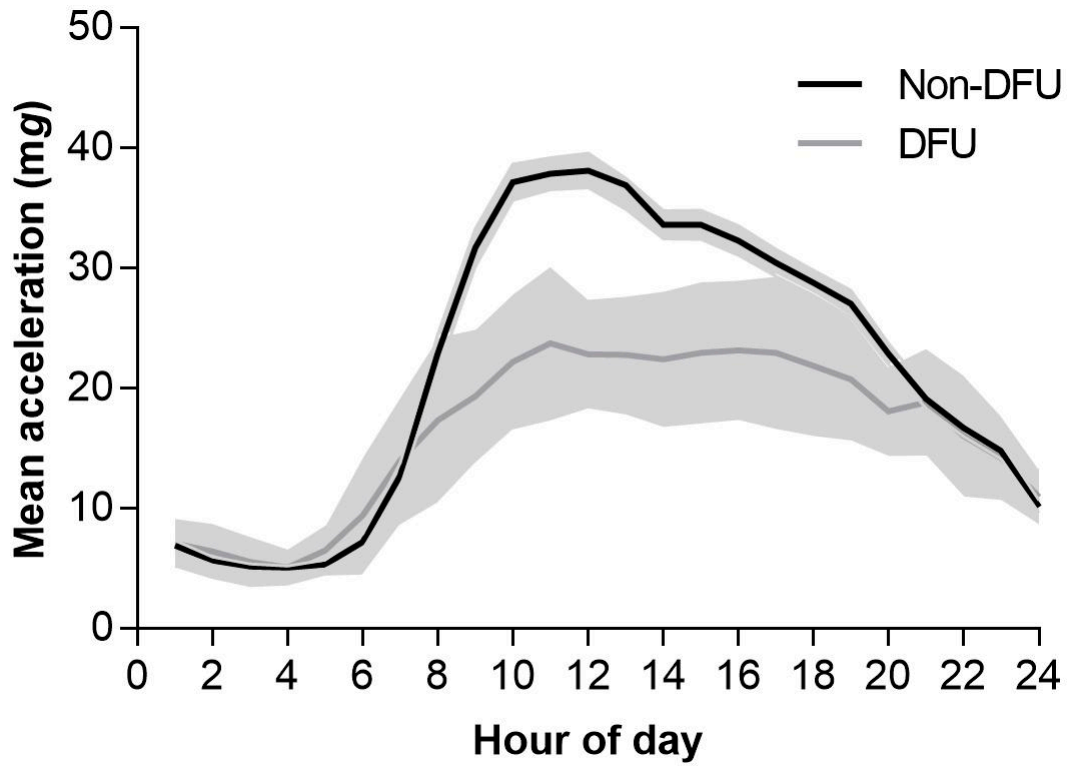
Mean hour-by-hour acceleration (95%CI) across the day by DFU status, adjusted for age, sex, and ethnicity.

**Figure 2:**
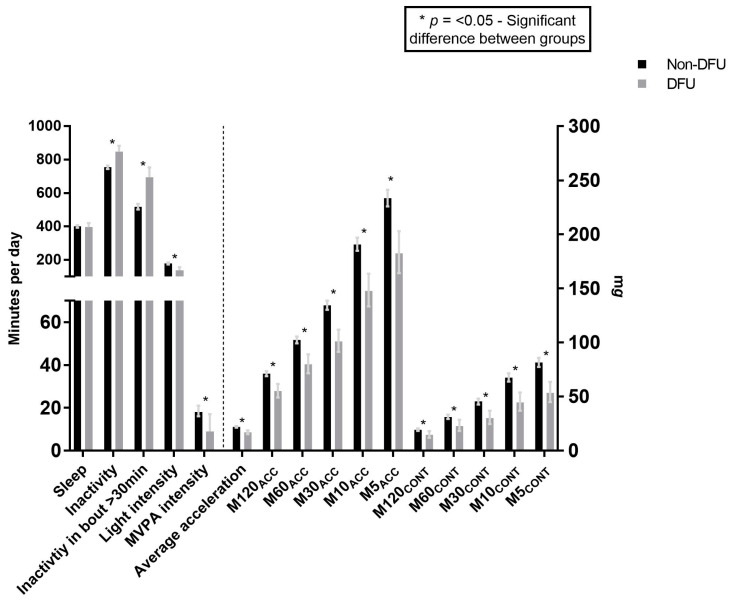
Marginal means (95% CI), adjusted for age, sex, BMI, ethnicity, presence of cardiovascular disease, diabetes duration and accelerometer wear time, presented for sleep duration, physical activity intensities, average acceleration, M120_CONT_, M60_CONT_, M30_CONT_, M10_CONT_, and M5_CONT_, M120_ACC_, M60_ACC_, M30_ACC_, M10_ACC_, and M5_ACC_ metrics in non-DFU and DFU groups.MVPA = Moderate to vigorous physical activity (>100 mg). Average acceleration = Proxy for volume of physical activity. M120_CONT_, M60_CONT_, M30_CONT_, M10_CONT,_ and M5_CONT_ = Intensity (acceleration) during the most active continuous 5, 10, 30, 60, and 120 min intervals. M120_ACC,_ M60_ACC,_ M30_ACC_, M10_ACC_, and M5_ACC_ = Intensity (acceleration) above which the most active 5, 20, 30, 60, and 120 min intervals are accumulated across the day.

The hour-by-hour acceleration profiles throughout the 24 h day for non-DFUs and DFUs are displayed in Figure 1. Average acceleration, a proxy for overall physical activity volume, was lower in those with DFUs compared to those with non-DFUs (21.9 mg [95%CI:21.2, 22.7] vs. 16.9 mg [15.3, 18.8] *p* ≤ 0.001; Figure 2). Those with DFUs also had a lower intensity gradient, indicating proportionally less time accumulated in higher-intensity activities (−2.741 [−2.763, −2.721] vs. −2.875 [−2.943, −2.807] *p* ≤ 0.001).

Non-DFU M5_CONT_ and M10_CONT_ were 81.6 mg (77.4, 85.9) and 67.6 mg (63.9, 71.5), respectively. It has been reported that a mean acceleration of 73 mg represents slow walking (3 km/h) [23] and 170 mg represents steady walking at 5 km/h [23], suggesting those with non-DFUs undertook sustained daily physical activity lasting 5–10 min that was consistent to a slow walking pace. However, in those with DFUs, the M5_CONT_ and M10_CONT_ were 53.4 mg (44.9, 63.6) and 44.5 mg (36.9, 53.7), respectively, suggesting that even slow walking was not sustained for 5 min in this population. (Figure 2).

The lower intensity of sustained (continuous) minutes of activity (MX_CONT_) in those with DFUs compared to those with non-DFUs was disproportionately greater than for accumulated minutes of activity (MX) (Table 3). For example, the % difference for the M5_CONT_ was 41.8% compared to 24.5% for M5_ACC_. The % difference between those with DFUs and non-DFUs was greater for all MX_CONT_ compared to the corresponding MX_ACC_ metrics.

Sleep durations were 400 min/day (393, 408) and 396 min/day (372, 421) for those with non-DFUs and DFUs, respectively (*p* = −612). Sleep efficiency was 86% (85, 87) for those with non-DFUs and 86% (83, 88) for those with DFUs (*p* = 0.749). The sleep midpoint (hh:mm) and sleep midpoint variability were 03:24 (03:15, 03:33) and 47 min/day (44, 50) for those with non-DFUs. The corresponding values for those with DFUs were 03:42 (03:12, 04:18) and 51 min/day (41, 64). Both sleep midpoint (*p* = 0.255) and sleep variability (*p* = 0.437) were non-significant between those with DFUs and non-DFUs.

Despite similar sleep durations, DFU accumulated more time inactive (848 min/day [812, 883] vs. 755 min/day [744, 765], *p* ≤ 0.001) and accumulated more of this time in prolonged bouts of inactivity (696 min/day [639, 754] vs. 518 min/day [501, 535], *p* ≤ 0.001; Figure 2). DFU also engaged in less light intensity (138 min/day [119, 157] vs. 179 min/day [173, 185], *p* ≤ 0.001) and MVPA (9 min/day [5, 17] vs. 18 min/day [16, 21], *p* ≤ 0.003); Figure 2).

## 4. Discussion

In this study, those with T2DM displayed high levels of inactivity with little activity at or above intensities, reflecting a steady walking pace. Activity profiles were consistent with previous research for those with chronic disease and who are impaired in comparison to the general population [26,27]. However, within this T2DM inactive population, time allocation to physical activity was particularly compromised in those with DFUs. Previous studies on those with DFUs have also consistently reported that people with DFUs are less physically active, with fewer steps per day reported compared to individuals without foot ulcers [15]. This study supports these findings of reduced activity in patients with DFUs whilst further contributing to the understanding of how physical activity profiles differ in this population. This study extends previous findings by applying recent developments in 24 h device-based measurement methods to DFUs [17]. Using this approach highlighted that the intensity of sustained activity was particularly impaired in those with DFUs, whilst sleep profiles were similar to a general population with type 2 diabetes [28]. 

The intensity of accumulated (MX_ACC_) and continuous (MX_CONT_) activity for both those with DFUs and non-DFUs reported are lower when compared to values previously reported for desk-based office workers, particularly for the DFU group [29]. Non-DFU MX_CONT_ and MX_ACC_ values were similar to those reported in individuals previously admitted to hospitals for COVID−19; however, DFU values were again lower [30]. The most striking difference between the two groups’ physical activity was in the continuous activity (MX_CONT_) metrics. The % difference between DFU and non-DFU in these metrics was greater compared to the total time accumulated (MX_ACC_) metrics. This highlights the importance of including an assessment of how physical activity is accumulated as well as how much activity is accumulated. Metrics that reflect the intensity of continuous physical activity or intervals of activity are easily derivable from accelerometers and allow for a greater understanding of physical activity profiles and how conditions, such as DFUs, impact physical activity. 

Previous research suggests a difference in average acceleration of 1 mg as the minimum clinically important difference (MCID) for health outcomes [31]. The difference between those with DFUs and non-DFUs was over four times greater than the MCID. The lower overall physical activity levels, along with a lower intensity of sustained movement, suggest compliance with offloading recommendations. However, it also highlights the need for adaptations to the physical activity recommendations in this population [32,33], given that the health benefits of physical activity, especially MVPA, are important in the management of diabetes [7]. Effective interventions for those with DFUs that promote increased physical activity whilst not compromising wound healing are therefore required. As the sustained activity was further limited in those with DFUs compared to non-DFUs, interventions should also ensure they are targeting this aspect of the physical activity profile. The current evidence suggests that weight bearing activity should be limited to ‘safe’ levels for patients with current DFUs [9] and then should be increased gradually, with close monitoring of the feet [34]. Offloading does not have to result in low physical activity levels, as alternative forms of activity are available that do not impede offloading or directly increase weight bearing. Non-weight-bearing seated physical activity could be one way of achieving clinically meaningful increases in physical activity and cardiorespiratory fitness, as has been shown through the use of arm ergometry in other populations with disability [35], with potential application for those with DFUs [32].

This study has some limitations. Offloading requirements were not reported for this cohort; this is likely to influence physical activity capabilities. The low sample size may limit generalisability. Wrist-worn accelerometers potentially do not capture all activities. However, they are ideally suited to the DFU population as they would capture any seated and/or offloaded activities undertaken, such as upper body ergometry. This is an important consideration for the interpretation of these results. DFU populations are often told to offload, but the measurement method used in this study would have captured alternative activities that they may have undertaken in place of this. However, there is still a very large difference in the physical activity level between those with DFUs and non-DFUs.

## 5. Conclusions

In conclusion, the overall volume of physical activity and the intensity of activity, particularly the intensity of sustained activity, but not sleep, are substantially lower in those with DFUs compared to non-DFUs. These findings build upon previous research into the activity levels of both those with non-DFUs and DFUs whilst applying recent advances in 24 h physical activity measurement and accelerometry methodology. The accelerometry metrics reported in this study provide a more detailed understanding of the 24 h physical activity profiles of both those with non-DFUs and DFUs. In particular, we report the finding that continuous activity is most impacted in those with DFUs. This underscores the need for tailored physical activity interventions that meet current guidelines of 150 min a week of at least moderate-intensity activity [7] whilst adhering to clinical offloading recommendations. This study should act as a stimulus for further research in larger and more representative samples.

## Figures and Tables

**Table 1:** Definition and description of the movement and sleep behaviour variables derived from accelerometers.

Variable	Definition and Description
Average acceleration	Reflects the volume or overall physical activity level in mg across the 24 h day.
Intensity gradient	Measure of overall distribution of activity intensities across the 24 h day [17]. In brief, it describes the negative curvilinear relationship between physical activity intensity and the time accumulated at that intensity during the 24 h day. The intensity gradient is always negative, reflecting the drop in time accumulated as intensity increases; a more negative (lower) gradient reflects a lower amount of time accumulated at mid-range and higher intensities, while a less negative (higher) gradient reflects more time spread across the intensity range.
Inactivity total	Time accumulated in a sedentary outside sleep window defined as accelerations lower than 40 mg [21].
Inactivity 30 min bouts	Time accumulated in inactive/sedentary intervals >30 min.
Light intensity activity	Time in light activity defined as time accumulated with accelerations between 40 and 100 mg.
Moderate to Vigorous activity (MVPA)	Time accumulated in moderate to vigorous activity, defined as time spent with accelerations >100 mg [23] in 1 min intervals.
M5_CONT_, M10_CONT_, M30_CONT_, M60_CONT_, M120_CONT_ Metrics	Acceleration threshold above which the most active continuous 5, 10, 30, 60, and 120 min bout of the daywas spent. This measure indicates that 75% of the most active X minutes were above this acceleration, i.e., if M30_CONT_ >100, then the majority of that 30 min period was spent in MVPA (>100 mg), with less than 25% of the time dropping below this threshold, e.g., stopping at traffic lights, tying shoelaces, etc.
M5_ACC_, M10_ACC_, M30_ACC_, M60_ACC_, M120_ACC_ Metrics	Acceleration above which a person’s most active X minutes (where X = number of minutes, 5–120) are accumulated across the day.
Sleep duration	The total time accumulated in minutes sleeping during the sleep window.
Sleep efficiency	Actual sleep within sleep window/sleep window duration.
Sleep midpoint	Time of middle of night period.
Sleep midpoint standard deviation	Standard deviation of middle of night period (mins/day).

**Table 2:** Participant characteristics.

	Non-DFU	DFU
N	561	34
Age (years)	64.3 ± 8.2	58.9 ± 8.9
Sex		
Male	361 (64.3%)	30 (88.2%)
Female	200 (35.7%)	4 (11.8%)
BMI (kg/m^2^)	31.0 ± 5.1	32.7 ± 4.8
Ethnicity		
White	472 (84.1%)	32 (94.1%)
Non-white	89 (15.9%)	2 (5.9%)
Diabetes duration (years)	10.3 ± 7.5	15.4 ± 9.6
Cardiovascular disease		
Yes	309 (55.1%)	14 (41.2%)
No	252 (44.9%)	20 (58.8%)
Mean accelerometer wear time (days)	6.9 ± 0.4	6.8 ± 0.7

**Table 3:** Estimated marginal means (95% CI) and % difference between groups for MX_CONT_ and MXA_CC_ metrics (mg).

	Non-DFU	DFU	*p* Value	% Difference
M5_CONT_	81.6 (77.4, 85.9)	53.4 (44.9, 63.6)	<0.001	41.8
M10_CONT_	67.6 (63.9, 71.5)	44.5 (36.9, 53.7)	<0.001	41.2
M30_CONT_	45.2 (42.6, 48.1)	30.1 (24.5, 36.9)	<0.001	40.1
M60_CONT_	31.1 (29.2, 33.2)	22.8 (18.2, 28.5)	0.001	30.8
M120_CONT_	19.3 (18.2, 20.5)	14.6 (11.9, 18.1)	0.003	27.7
M5_ACC_	233.4 (225.8, 241.2)	182.5 (164.1, 203.0)	<0.001	24.5
M10_ACC_	190.7 (184.8, 196.9)	147.7 (133.4, 163.6)	<0.001	25.4
M30_ACC_	134.4 (130.2, 138.6)	101.1 (91.4, 111.9)	<0.001	28.3
M60_ACC_	102.4 (99.2, 105.6)	79.8 (71.5, 89.1)	<0.001	24.8
M120_ACC_	71.2 (68.9, 73.6)	55.0 (49.0, 61.7)	<0.001	25.5

## Data Availability

The data that support the findings of this study are not openly available due to containing information that could compromise research participant privacy/consent. Requests for participant-level quantitative data and statistical codes should be made to the corresponding author. Data requests will be put forward to members of the original trial management team, who will release data on a case-by-case basis.
